# Models for 31-Mode PVDF Energy Harvester for Wearable Applications

**DOI:** 10.1155/2014/893496

**Published:** 2014-07-09

**Authors:** Jingjing Zhao, Zheng You

**Affiliations:** ^1^Collaborative Innovation Center for Micro/Nano Fabrication, Device and System, Tsinghua University, Beijing 100084, China; ^2^State Key Laboratory of Precision Measurement Technology and Instrument, Tsinghua University, Beijing 100084, China; ^3^Department of Precision Instrument, Tsinghua University, Beijing 100084, China

## Abstract

Currently, wearable electronics are increasingly widely used, leading to an increasing need of portable power supply. As a clean and renewable power source, piezoelectric energy harvester can transfer mechanical energy into electric energy directly, and the energy harvester based on polyvinylidene difluoride (PVDF) operating in 31-mode is appropriate to harvest energy from human motion. This paper established a series of theoretical models to predict the performance of 31-mode PVDF energy harvester. Among them, the energy storage one can predict the collected energy accurately during the operation of the harvester. Based on theoretical study and experiments investigation, two approaches to improve the energy harvesting performance have been found. Furthermore, experiment results demonstrate the high accuracies of the models, which are better than 95%.

## 1. Introduction

As a power supply, an electrochemical battery is necessary to wearable electronics, which is one focus of wearable electronics. But two obvious disadvantages of these batteries, limited energy storage capability and potential damage to environment, have limited further development and applications of wearable electronics. Many efforts have been focused on wearable energy harvesters, which can harvest the mechanical energy dissipated from human motion to supply renewable and clean energy [1]. There have been several concepts of harvesting energy from human motion with varied mechanisms to be studied [2–13], including electromagnetic [5–8], electrostatic [9], thermoelectric [10], and piezoelectric harvesters [11–13]. Among them, piezoelectric energy harvester can convert mechanical energy into electric power directly, resulting in a more compact and simpler structure. Lead zirconate titanate (PZT) and PVDF are two common functional materials for piezoelectric energy harvesters. Compared with rigid, brittle, and heavy PZT, PVDF has considerable flexibility, good stability, and easiness to handle and shape [4]. Considering that human motion is characterized by high amplitude and low frequency (less than 5 Hz typically [14, 15]), the advantages presented above make PVDF more appropriate for wearable applications where flexibility is necessary, compared with PZT. PVDF has been designed for wearable energy harvesters implemented in shoes [16–19], bags [20, 21], and clothing [22, 23]. Almost all the reported PVDF energy harvesters are based on 31-mode, because 31-mode can easily yield larger strain by smaller forces in comparison with 33-mode.

As discussed above, 31-mode PVDF energy harvester for wearable applications has been studied widely. But there is still a lack within the general theoretical models for design, making it hard to analyze and optimize the energy harvester. So this paper focuses on a series of models, including basic model, resistive load model, capacitive load model, and energy storage model, which can give theoretical support to this kind of harvester. Design and analysis of the device with accuracy and facility can be realized by using the models. Experiments show that the models were accurate with errors less than 5%. Furthermore, with the help of the models and experimental results, it has been found that two approaches can improve the performance of energy harvesting.

## 2. Models for 31-Mode PVDF Energy Harvester

### 2.1. PVDF Energy Harvester Unit

**Figure 1:**
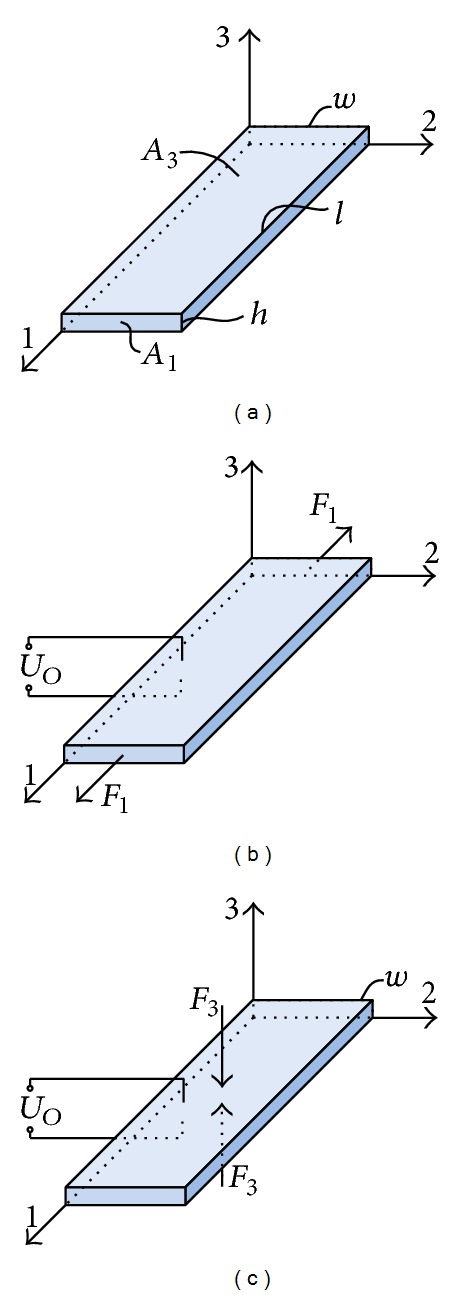
(a) A PVDF energy harvester unit, (b) 31-mode, and (c) 33-mode.

**Figure 2:**
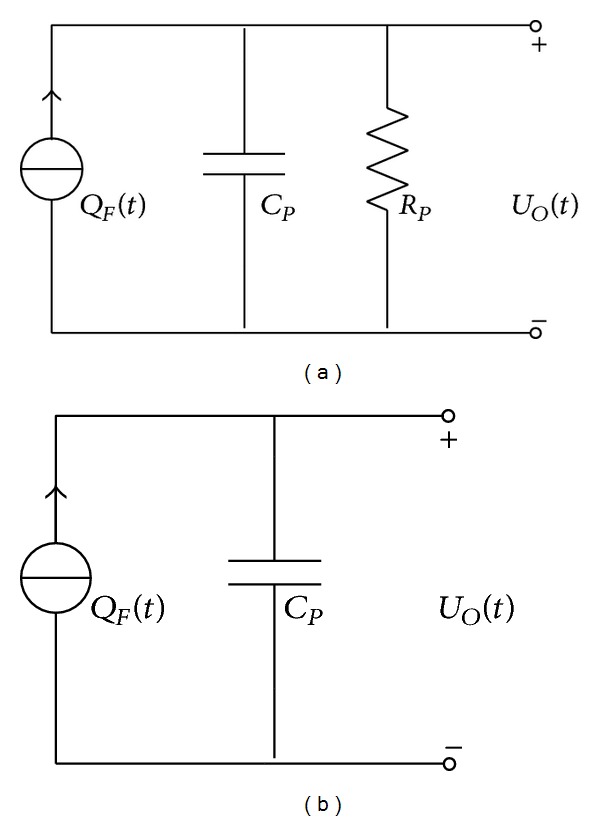
(a) The equivalent circuit for a PVDF energy harvester unit, (b) The ideal equivalent circuit.

A PVDF energy harvester unit studied in this paper is shown in Figure 1(a), which is made of a layer of PVDF film. A cartesian coordinate system is established to identify the directions within the PVDF unit. 1-axis and 3-axis are mechanical stress direction and polarization direction during PVDF fabrication, respectively. Electrodes are placed on the surfaces perpendicular to 3-axis and *U*_*O*_ is the output voltage between the two electrodes. *l*, *w*, and *h* are length, width, and thickness of the PVDF unit. *A*_3_ is the area of one 3-axis surface or one electrode, and *A*_1_ is the area of cross-section along 1-axis. The PVDF energy harvester unit can be used individually, or several units are stacked and connected in series or in parallel for higher output voltage or current.  Figure 2(a) shows the typical equivalent circuit used to present the operation of the unit. *Q*_*F*_ is an equivalent charge generator representing the piezoelectric charges. *R*_*P*_ and *C*_*P*_ are the resistance and capacitance of the PVDF unit, and *U*_*O*_ is the output voltage. *R*_*P*_ is extremely large with little current flowing, therefore considering *R*_*P*_ infinite. Thus, the equivalent can be simplified as shown in Figure 2(b).

31-mode and 33-mode are two common modes in which piezoelectric energy harvesters operate generally. A force is applied along 1-axis then charges accumulate on the 3-axis electrodes when the harvester unit works in 31-mode, and the applied force direction parallels 3-axis then charges generated on the 3-axis electrodes in 33-mode, as shown in Figures 1(b) and 1(c). Benefiting from *A*_1_ which is much smaller than *A*_3_, the PVDF unit operated in 31-mode can gain a far larger strain comparing to the operation in 33-mode, when the applied forces have the same amplitude. Therefore, more charges can be produced in 31-mode. Furthermore, the forces along 1-axis are very common in wearable applications, which can be produced by limbs motion [23]. Based on the analysis above, the models for PVDF energy harvester operated in 31-mode are more valuable and this paper focuses on them.

**Figure 3:**
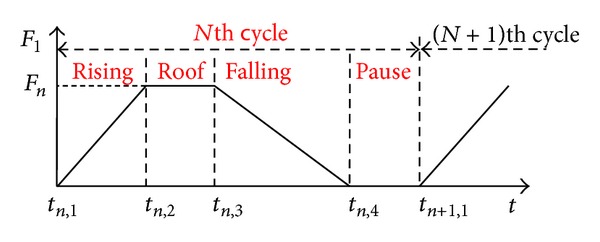
The abstract model of *F*_1_ which can be divided into four stages during one cycle.

The force along 1-axis produced by human motion (*F*_1_) is an alternating force with a low frequency less than 5 Hz. The abstract model of *F*_1_ is shown in Figure 3, and during one cycle the force can be divided into four stages: rising, roof, falling, and pause. *F*_*n*_ is the amplitude of the *N*th cycle, which is a key parameter for calculating the quantity of stored energy or charges. Basic model, resistive model, capacitive load model, and energy storage model of the PVDF unit are established step by step as below.

### 2.2. Basic Model

The basic model describes the piezoelectric performance of a PVDF unit operated in 31-mode. And the piezoelectric constitutive equations for 31-mode are

(1) S1=s11ET1+d31E3,D3=d31T1+ε33TE3,

where *S*_1_ is the strain, *s*_11_^*E*^ is the elastic compliance under constant electric field, *T*_1_ is the stress, *d*_31_ is the piezoelectric constant, *E*_3_ is electric field, *D*_3_ is the electric displacement, and *ε*_33_^*T*^ is the permittivity under constant stress. *F*_1_ is produced by human motion and dynamic, leading to inertial force and damping force in PVDF unit. Considering that the frequency of the human motion is low enough and the weight of PVDF unit is light, the values of inertial and damping forces can be neglected. The constitutive equations can be simplified by the following relationships [20]:

(2) D3(t)=Q3(t)A3=Q3(t)lw,E3(t)=UO(t)h,T1(t)=F1(t)A1=F1(t)wh,S1(t)=y(t)l,

where *Q*_3_ is the free charges collected on the electrodes (equal to the charges flowing to the external circuit), *U*_*O*_ is the output voltage, *F*_1_ is the dynamic force applied on PVDF along 1-axis, and *y* is the displacement along 1-axis. The piezoelectric constitutive equations can be rewritten as

(3) F1(t)=F11(t)+F12(t),QF(t)=d31lhF1(t)=Q3(t)+CPUO(t),F11(t)=YA1ly(t),F12(t)=d31YwUO(t),CP=ε33A3h,

where *Y* is the PVDF elastic modulus, *Q*_*F*_ is the generated charges, *C*_*P*_ is the capacitance of PVDF unit, *F*_11_ is the deformation force, and *F*_12_ is the force caused by the secondary converse piezoelectric effect. *F*_12_ reaches maximum when the PVDF unit is open without no load (*Q*_3_ = 0), and the maximum value is far less than *F*_1_, as expressed in (4). Hence, *F*_12_ can be neglected and (3) can be simplified as (5)


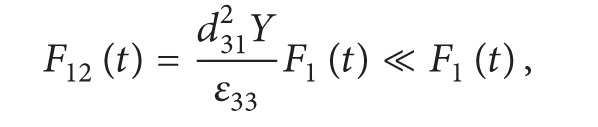

(4)



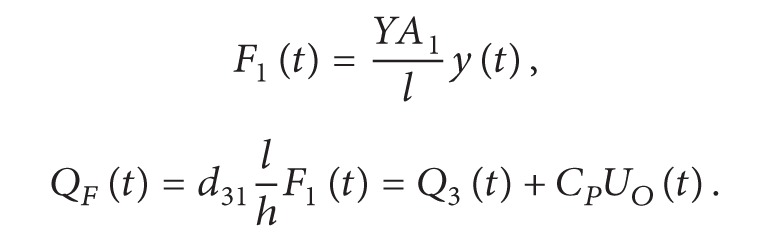

(5)

The model, expressed by (5), describes the piezoelectric performance of PVDF unit operated in 31-mode, and it also acts as the basic model in order to give the fundamental supports to other models discussed below.

### 2.3. Resistive Load Model

**Figure 4:**
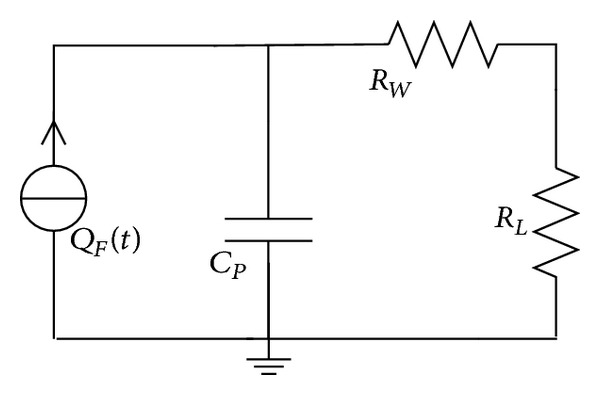
PVDF unit is connected with a resistive load.

The schematic diagram of the equivalent circuit is transferred as shown in Figure 4, when the PVDF unit is terminated with a resistive load *R*_*L*_. *R*_*W*_ presents the wire resistance which is much lower than *R*_*L*_ typically and can be ignored. The applied force is defined in (6), consisting of a sinusoidal force component and a preload force component for convenience of experimental verifications. The amplitude of the preload force is greater than that of the sinusoidal one in order to keep the film unit tensional. Equation (5) can be rewritten as (7). The output voltage is given in (8) and the power output can be calculated by *U*_*O* ^2^_/*R*_*L*_. The resistive load model offers an approach to test the piezoelectric performance of PVDF unit operating in 31-mode by measuring the output voltage, and it can further evaluate the generating capacity by calculating the output power


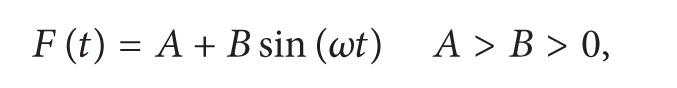

(6)



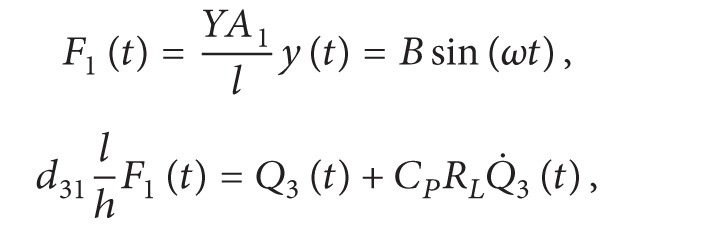

(7)



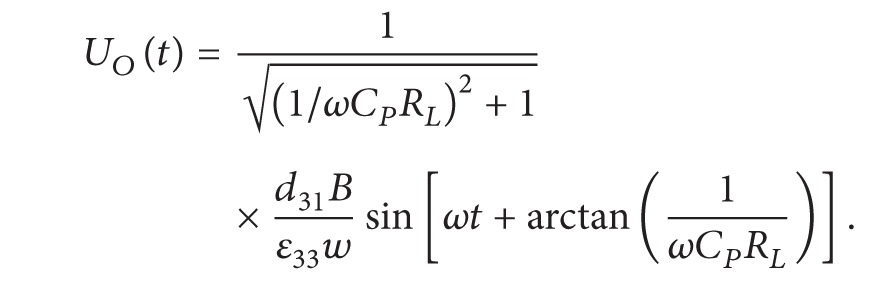

(8)



### 2.4. Capacitive Load Model

**Figure 5:**
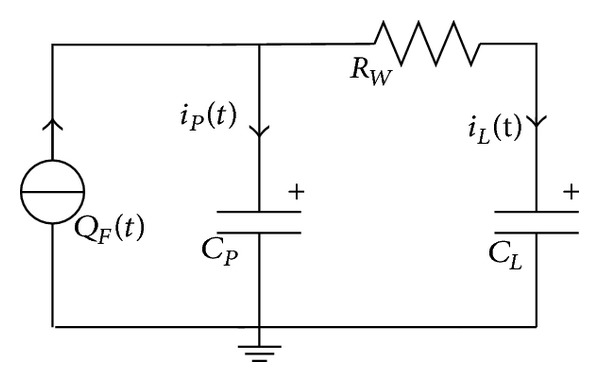
PVDF unit with capacitive load.

**Figure 6:**
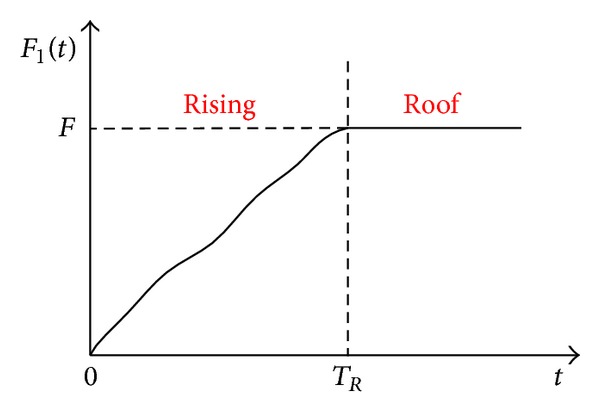
The applied force is composed of rising and roof stages.

Figure 5 shows the equivalent circuit of the capacitive load model in which the PVDF unit is connected with a capacitor to store the generated charges. The applied force is composed of rising and roof stages expressed in Figure 3 in order to keep the output voltage direction unchanged. The applied force is shown in Figure 6, where *T*_*R*_ is the force rise time, *F* is the amplitude value, *C*_*L*_ is the storage capacitor, and *C*_*P*_ is the capacitance of PVDF unit.

By using Kirchhoff's voltage law and Kirchhoff's current law, (9) can be given as

(9) ∫0tiL(t)dt+∫0tiP(t)dt=QF(t),∫0tiP(t)dtCP=∫0tiL(t)dtCL+RWiL(t)+QL(0)CL,QL(t)=∫0tiL(t)dt+QL(0),QF(t)=d31lhF1(t),0≤t≤TR,

where *R*_*W*_ is the wire resistance, *i*_*P*_ and *i*_*L*_ are branch currents, *Q*_*L*_ is the charges stored in *C*_*L*_, and *Q*_*L*_(0) is the initial value. Equation (10) is obtained by Laplace transform

(10) QL(s)=CLQF(s)sCLCPRW+(CL+CP)+CL2CPRWUL(0)sCLCPRW+(CL+CP) +CL2UL(0)s2CLCPRW+s(CL+CP).

The inverse Laplace transform of (10) is (11). *Q*_*L*1_ is the zero input response and *Q*_*L*2_ is the zero state response. Consider

(11) QL(t)=QL1(t)+QL2(t) (0≤t≤TR),QL1(t)=CLQF(t)CL+CP−CLe−t/τ∫0tet/τdQF(t),QL2(t)=e−t/τQL(0)+CLQL(0)CL+CP(1−e−t/τ),τ=CLCPRWCL+CP,

where *τ* is the time constant. Typically, the wire resistance *R*_*W*_ approaches zero, and *C*_*L*_ is much greater than *C*_*P*_. Hence, *τ* is approximately equal to zero and can be neglected when comparing to *T*_*R*_. Equation (11) can be simplified to

(12) QL(t)=QL1(t)+QL2(t),QL1(t)≈CLQF(t)CL+CP,QL2(t)≈CLQL(0)CL+CP,0≤t≤TR.

When the applied force *F*_1_ reaches the amplitude *F*, the charges on the *C*_*P*_ and *C*_*L*_ can be given by (13) according to the law of conservation of charge:

(13) QL(t)=CLCL+CP[QF(TR)+QL(0)],QP(t)=CPCL+CP[QF(TR)+QL(0)],QF(TR)=d31lhF.

The numerical relationship is established between the quantity of stored charge and the amplitude of applied force, which gives supports for the energy storage model.

### 2.5. Energy Storage Model

**Figure 7:**
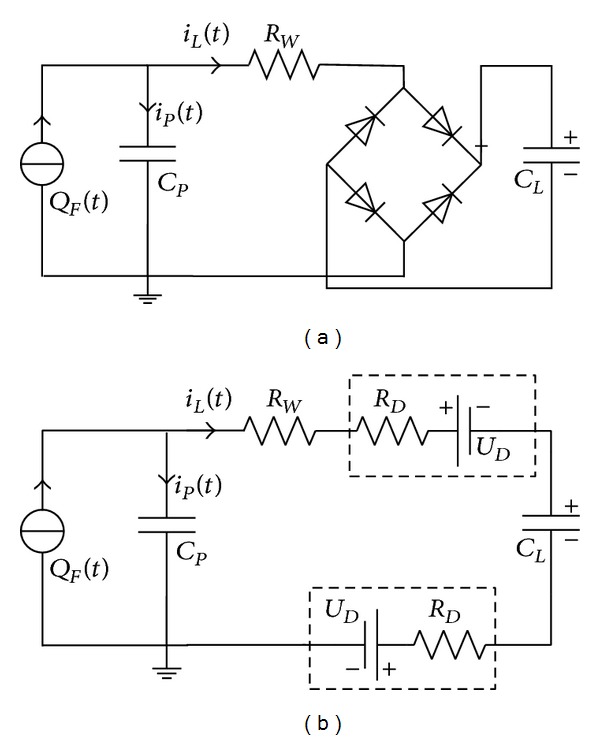
(a) The circuit for energy storage and (b) the equivalent circuit when bridge rectifier conducting.

This model can predict the quantities of the collected charges and stored energy and help to design and optimize a 31-mode PVDF energy harvester. The energy storage circuit with a PVDF unit is shown in Figure 7(a). The circuit is widely used for piezoelectric harvester, consisted of a bridge rectifier and a storage capacitor. The rectifier alternates the generated current, and the charges accumulate on the storage capacitor *C*_*L*_. When two diodes are conducting, the equivalent circuit is presented in Figure 7(b), which is similar to the circuit discussed in Section 2.3. *U*_*D*_ is the diode forward voltage, and *R*_*D*_ is the diode forward resistance. The time constant *τ* is defined as

(14) τ=CLCP(RW+2RD)CL+CP.

**Figure 8:**
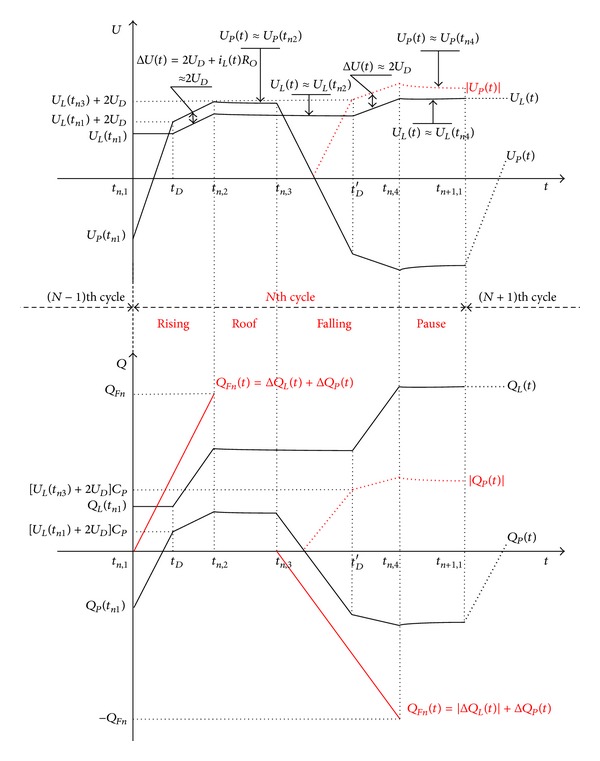
The charging process of *C*_*L*_ during one cycle.

The increase of the charges stored in *C*_*L*_ during one cycle is determined by the force amplitude of every cycle according to the analysis in Section 2.4. Therefore, using the abstract force in Figure 3 as the applied force to analyze the charging process is reasonable. The force period is far greater than *τ*; therefore the delay between *Q*_*L*_(*t*) and *Q*_*F*_(*t*) can be neglected. The equations obtained in Section 2.4 can be used here. The four stages in the *N*th cycle are analyzed below, and the charging process of *C*_*L*_ is shown in Figure 8.

*Rising Stage (t*_*n*,1_~*t*_*n*,2_ *).* Rising stage can be divided into two parts by *t*_*D*_ which is the first rectifier turn-on time. The voltage on *C*_*P*_ is lower than rectifier threshold voltage and the generated charges accumulate in *C*_*P*_ before *t*_*D*_. After *t*_*D*_, the current *i*_*L*_ flows through the rectifier and charges are stored in *C*_*P*_ and *C*_*L*_. At the moment *t*_*n*,2_, the force stops rising and reaches the amplitude *F*_*n*_ of the *N*th cycle. This stage can be presented by the following equations, where *U*_*P*_ is the voltage across *C*_*P*_, and *U*_*L*_ is the voltage across *C*_*L*_:

(15) tn1≤t≤tD:{UL(t)=UL(tn1)UP(t)=UP(tn1)+QFn(t)CP,t=tD:{UP(tD)=UL(tn1)+2UDQFn(tD)=[UL(tn1)+2UD−UP(tn1)]CP,tD≤t≤tn2:{UL(t)=UL(tn1)+QFn(t)−QFn(tD)CP+CLUP(t)=UL(tn1)+2UD+QFn(t)−QFn(tD)CP+CL,t=tn2:{QL(tn2)=CL[QFn(tn2)+QL(tn1)+QP(tn1)]CP+CL −2CLCPUDCP+CLQP(tn2)=CP[QFn(tn2)+QL(tn1)+QP(tn1)]CP+CL +2CLCPUDCP+CLQFn(tn2)=d31lhFn.

*Roof Stage (t*_*n*,2_~*t*_*n*,3_ *).* During roof stage, the applied force keeps unchanged, so no charge is generated and the rectifier turns off. The leakage current of capacitors is quite low and the stage's duration is short. It can be concluded that no charge on *C*_*P*_ and *C*_*L*_ leaks away, so equations can be given as

(16) tn2≤t≤tn3:{QL(t)=QL(tn2)QP(t)=QP(tn2).

*Falling Stage (t*_*n*,3_~*t*_*n*,4_ *).* The input force decreases to zero from *F*_*n*_. This stage is similar to the rising stage and can be divided into two parts by *t*_*D*_′, the rectifier turn-on time during this stage. By referring to the analysis of rising stage, the charging process can be presented by the following equations:

(17) tn3≤t≤tD′:{UL(t)=UL(tn3)UP(t)=UP(tn3)+QFn(t)CP,t=tD′:{UP(tD′)=−[UL(tn3)+2UD]QFn(tD′)=−[UL(tn3)+2UD+UP(tn3)]CP,tn3≤t≤tn4:{UL(t)=UL(tn3)−QFn(t)−QFn(tD′)CP+CLUP(t) =−[UL(tn3)+2UD−QFn(t)−QFn(tD′)CP+CL],t=tn4:{QL(tn4)=CL[QL(tn3)−QFn(tn4)−QP(tn3)]CP+CL −2CLCPUDCP+CLQP(tn4)=−CP[QL(tn3)−QFn(tn4)−QP(tn3)]CP+CL −2CLCPUDCP+CLQFn(tn4)=−d31lhFn.

*Pause Stage (t*_*n*,4_~*t*_*n*+1,1_ *).* No force is applied on PVDF until the (*N* + 1)th cycle begins. The charges on *C*_*P*_ and *C*_*L*_ remain constant for the same reasons presented in stay stage. Consider

(18) tn4≤t≤t(n+1)1:{QL(t)=QL(tn4)QP(t)=QP(tn4).

As discussed above, the charges are generated and collected on *C*_*L*_ during rising and falling stages, and the applied force keeps constant with no charge generated in roof and pause stages. Recursion formulae of *Q*_*L*_ and *Q*_*P*_ can be derived and be presented by

(19) QL,0=QL(N),QP,0=QP(N),QL,1=t1(QF+QL,0+QP,0)−2t1UDCP,QP,1=t2(QF+QL,0+QP,0)+2t1UDCP,QL,2=t1(QF+QL,1−QP,1)−2t1UDCP,QP,2=−t2(QF+QL,1−QP,1)−2t2UDCL,t1=CL(CL+CP),t2=CP(CL+CP),QF=Fnd31lh,QL(N+1)=QL,2,QP(N+1)=QP,2,

where *N* is the cycle number, *Q*_*L*_(*N*) is the quantity of the charges on *C*_*L*_ at the end of the *N*th cycle, *Q*_*P*_(*N*) is the quantity of charges on *C*_*P*_ at the end of the *N*th cycle, *F*_*n*_ is the force amplitude in the *N*th cycle, and *t*_1_ and *t*_2_ are two defined parameters for simplification. *Q*_*L*,0_ and *Q*_*P*,0_ are the initial electric quantities of one cycle, *Q*_*L*,1_ and *Q*_*P*,1_ are the electric quantities when the applied force reaches *F*_*n*_, and *Q*_*L*,2_ and *Q*_*P*,2_ are the electric quantities when the force returns to its initial value. If the force amplitude in each cycle can be acquired by test or calculation, the recursion formulae can be utilized to estimate the charges collected on storage capacitor. In some cases, the force amplitude in each cycle is a constant value *F* and *C*_*P*_ as well as *C*_*L*_ stores no charge initially; then (19) can be rewritten below as

(20) QL(N+1)=A1B+A+−1A+n−A1B+A+−1+B1,QP(N+1)=A2B+A+−1A+n−A2B+A+−1+B2,

(21) A1=(t1−t2)t1,A2=(t2−t1)t2,B1=−4t12UDCP−2t1UDCP+2t12QF,B2=4t1t2UDCP−2t1UDCP−2t1t2QF,A+=A1+A2=(t1−t2)2,B+=B1+B2=2QF(t1−t2)t1−8t12UDCP,QF=Fd31lh.

It is known that *C*_*L*_ is much greater than *C*_*P*_, and *U*_*D*_ is not more than 0.7 V; thus *t*_1_ is approximately 1 and *t*_2_ is close to 0. And the approximate expression of (20) is given below as

(22) QL(N+1)≈2QF+2QF1−A+N1−A+,QP(N+1)≈0.

According to (20) and (22), it is shown that *C*_*P*_ and *C*_*L*_ are main factors that influence the charge collection, while *U*_*D*_ has little influence on *Q*_*L*_, so it is not an important parameter that needs selecting carefully. Hence, researchers can focus on key parameters by analyzing the energy storage model. Besides, *Q*_*L*_ is approximately proportional to *Q*_*F*_ while *Q*_*F*_ is proportional to the applied force amplitude; therefore *Q*_*L*_ is approximately proportional to the force amplitude. In reality, *C*_*L*_ is far larger than *C*_*P*_; therefore most of the generated charges are transferred to *C*_*L*_. The charge increment per cycle declines as the cycle number increases, as shown in (23). The charge transfer efficiency *η*_*QL*_ is defined in (24). The energy stored on *C*_*L*_ is given in (25), and (26) presents the energy increment per cycle. Consider

(23) ΔQL(N)=QL(N)−QL(N−1)={A1B+A+N−1N≠1B1≈2QFN=1,

(24) ηQL(N)=ΔQL(N)2QF,

(25) EL(N)=QL(N)22CL,

(26) ΔEL(N)=EL(N)−EL(N−1).

The energy storage model is based on the energy storage circuit shown in Figure 7. The recursion formulae of stored charges are presented by (19), which can predict the charge quantity when the amplitude of applied force in each cycle is known. If the applied force keeps constant, the energy storage model can be presented by (20). Several parameters shown in (23)~(25) can evaluate the energy harvesting performance.

### 2.6. Two Approaches to Improve Energy Harvesting Performance

**Figure 9:**
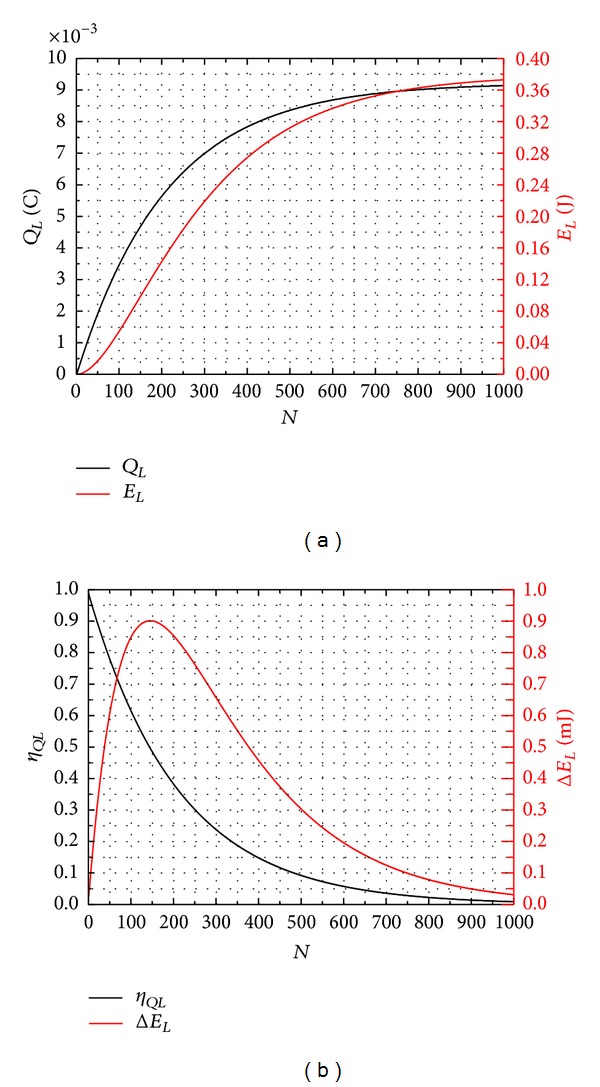
(a) The charges and energy stored on storage capacitor versus the cycle number. (b) The charges transfer efficiency and energy increment per cycle versus the cycle number.

On the basis of the energy storage model, two approaches to improve energy harvesting performance are derived. The first approach is that the voltage on storage capacitor should to be kept low for high energy storage efficiency. Several parameters of the energy storage model are assigned to detail for convenience to clear analysis and easy demonstration. Consider *C*_*L*_ = 112 *μ*F, *C*_*P*_ = 133 nF, *Q*_*F*_ = 43.3 *μ*C, and *U*_*D*_ = 0.53 V; then *Q*_*L*_, *η*_*QL*_, *E*_*L*_, and Δ*E*_*L*_ are simulated in Figure 9. Apparently, *η*_*QL*_ decreases exponentially as the cycle number *N* increases. Δ*E*_*L*_ first increases and then decreases with an increasing *N*. It is because that as the voltage on *C*_*L*_ increases, more charges are dissipated on *C*_*P*_. The stored energy on *C*_*L*_ should be moved to another energy storage device, like a battery, or consumed by load timely before Δ*E*_*L*_ begins to decrease or *η*_*QL*_ is lower than a certain value (85% for instance). After transferring the stored energy, the voltage on *C*_*L*_ will decrease and *C*_*L*_ can store energy with a high efficiency again. In conclusion, reducing the voltage across the storage capacitor is a viable approach for high energy harvesting performance.

The second approach is to improve the generated charges *Q*_*F*_. The electrical connection configuration of PVDF units affects *Q*_*F*_ considerably, and three common configurations are studied. The first configuration is one *h* thick PVDF unit, and the other two configurations are *n* layers of *h*/*n* thick PVDF units that are stacked and wired in parallel and series. In the three cases, PVDF units have the same length, width, and the total thickness. The generated charges of the three cases can be given by (27) and the PVDF equivalent capacitance is expressed by (28), where *S* means single PVDF unit, *nP* means *n* PVDF units in parallel, and *nS* means *n* PVDF units in series. Obviously, thin PVDF units with a parallel connection can generate most charges, proving that connecting thin PVDF units in parallel is an effective approach to improving the energy harvesting performance. Consider

(27) QF,S=d31lhF,QF,nP=nd31lhF=nQF,S,QF,nS=d31lhF=QF,S,

(28) CP,S=ε33A3h,CP,nP=nε33A3h/n=n2CP,S,CP,nS=1nε33A3h/n=CP,S.

## 3. Experiments

**Figure 10:**
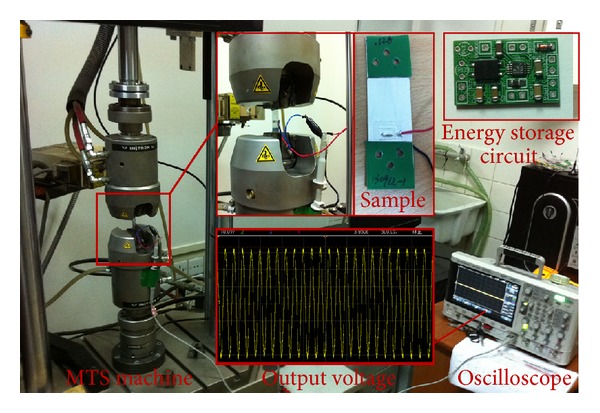
Testing system.

Two experiments were performed to verify the models and approaches established above. The testing system for model verification is shown in Figure 10. The PVDF samples consisted of several PVDF energy harvester units which were wired in series or parallel. The PVDF units were 70 mm × 20 mm in size, with sputter-deposited aluminum layer electrodes of 30 mm × 20 mm. The mechanical and electrical properties of the PVDF films made into the units were listed in Table 1. The sample capacitance *C*_*P*_ was measured by a digital LCR meter (Agilent E4980, at 20 Hz). The characteristics of the PVDF samples are listed in Table 2. A material testing system machine (MTS, INSTRON-8874) was run by load control to apply the forces defined in (6) on the PVDF samples. The output voltage of each sample was measured by an oscilloscope (DSOX2024A).

## 4. Results and Discussions

**Figure 11:**
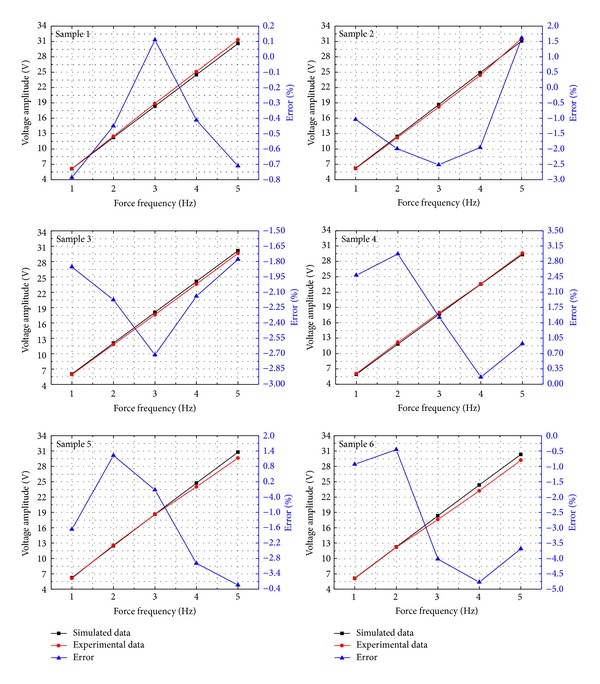
Experimental and simulated results of the output voltage amplitude of samples 1~6.

To test the basic model and resistive load model, the experiments were performed by the MTS machine with samples 1~6. The force frequency ranged from 1 Hz to 5 Hz because of human motion frequency of about 1~5 Hz. Samples 1~3 were 500 *μ*m thick and the applied force was (100 + 80sin*ωt*)*N*, and samples 4~6 were 100 *μ*m thick with the applied force of (40 + 20sin*ωt*)*N*. During testing, the output of a sample was terminated into the oscilloscope to measure the output voltage. The internal resistance of the oscilloscope was 10 MΩ, serving as a resistive load, and the output voltage amplitudes were simulated by (8).  Figure 11 compares the experimental and simulated results of the output voltage amplitudes of samples 1~6. The six samples were tested at 1~5 Hz, and all the results of simulation were in good agreement with the ones from the experiment measurements with errors less than 5%. According to (8), (29) can be given. It shows that samples 1~3 can produce approximately the same voltage as that samples 4~6 can produce, which is also demonstrated in Figure 11. In conclusion, the resistive load model is capable of predicting the output voltages of different samples accurately, indirectly proving that the basic model is accurate. Consider

(29) UAmplitude, Sample 1~3UAmplitude, Sample 4~6≈CP, Sample 4~6CP, Sample 1~3·BSample 1~3BSample 4~6=4CP, Sample 4~6CP, Sample 1~3≈1.

**Figure 12:**
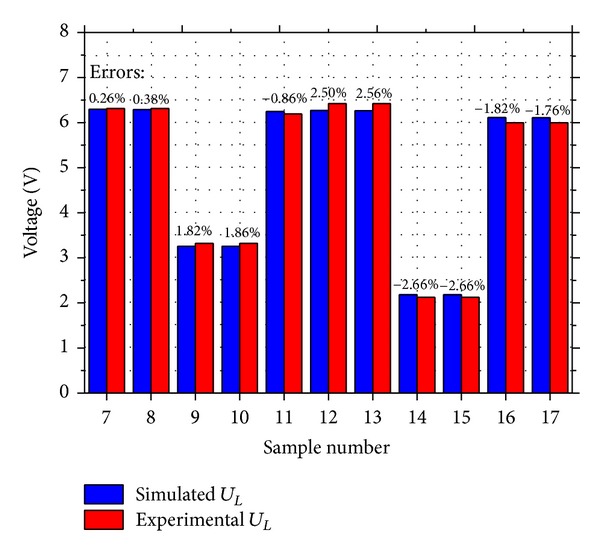
The experimental and simulated data of *U*_*L*_ after the PVDF samples 7~17 had been operated by the applied force (50 + 30sin4*πt*)*N* for 1 min.

The results of PVDF samples 7~17 were presented in Figure 12 to verify the capacitive load model and energy storage model. Samples 7~17 consisted of 1~3 layers of 100 *μ*m PVDF energy harvester units and the units were wired in series or parallel. The energy storage circuit shown in Figure 7 was used to store the generated charges on energy storage *C*_*L*_ of 11.3 *μ*F. The samples were operated by the applied force (50 + 30sin4*πt*)*N*. After one sample kept working for 1 min (or 120 cycles), the voltage on the storage capacitor *U*_*L*_ was measured to calculate the stored energy and charges. The simulated values of the voltage on *C*_*L*_ were calculated by (20) with *Q*_*F*_ predicted by (27). The samples had three connection configurations. *Q*_*F*_ for samples of one unit and several units in parallel was 0.153 *μ*C, *Q*_*F*_ for samples of two units in series was 0.0756 *μ*C, and *Q*_*F*_ for samples of three units in series was 0.051 *μ*C. In Figure 12, the simulated and experimental results of the sample were presented, and the errors were listed. There was great consistency between the experimental data and theoretical data with errors of 3%, which demonstrated that the capacitive load model and the energy storage model were accurate enough to describe PVDF energy harvester units. Obviously, the samples of PVDF units wired in parallel generated more charges and energy than the samples of PVDF units in series did. For example, the samples of two units in parallel (sample 9 and sample 10) produced about twice as many charges as the samples of two units in series (samples 11~13) did. The results were consistent with the theoretical investigation derived from (27).

## 5. Conclusions

This paper establishes a series of models for 31-mode PVDF energy harvester for wearable applications. Basic model presents the operation of PVDF with 31-mode. Resistive load model can test the performance of 31-mode PVDF energy harvester and predict harvested energy quantity. Capacitive load model describes the charge distribution. And energy storage model provides a way to calculate the stored charges and energy. Experiment results demonstrate the accuracies of the series of models that are better than 95% for every case tested. In order to improve and optimize the harvester, two approaches have been found through theoretical analysis and experimental investigation. Firstly, it is necessary to timely reduce the voltage across the energy storage device (a capacitor for instance). Second approach is that thin PVDF energy harvester units wired in parallel can generate more charges and improve the collected energy. The models and the approaches can serve as general rules for design and analysis of 31-mode PVDF energy harvester for wearable applications.

## Figures and Tables

**Table 1:** Properties of the PVDF materials.

Material property	Symbol	Value
Relative permittivity	*ε*_*r*_	9.5 ± 1
Resistivity	*ρ*_*r*_	10^11^ Ω*·*m
Mass density	*ρ*	1.78 g/cm^3^
Piezoelectric constant	*d*_31_	17 × 10^−12^ C/N
Elastic modulus	*Y*	2500 MPa
Planar Poisson's ratio	*υ*	0.3
Yield strength	*σ*_*s*_	45~55 MPa

**Table 2:** PVDF samples for verifying the models.

Sample number	*C*_*P*_ (nF)	Thickness (*μ*m)	Connection configuration
1	0.127	500	Single unit
2	0.129		
3	0.125		

4	0.492	100	Single unit
5	0.517		
6	0.510		

7	0.493	100	Single unit
8	0.515		

9	0.272	100 × 2	2 units in series
10	0.287		

11	1.095	100 × 2	2 units in parallel
12	1.038		
13	1.093		

14	0.184	100 × 3	3 units in series
15	0.182		

16	1.480	100 × 3	3 units in parallel
17	1.533		
